# Bruton Tyrosine Kinase Inhibition and Its Role as an Emerging Treatment in Pemphigus

**DOI:** 10.3389/fmed.2021.708071

**Published:** 2021-08-10

**Authors:** Aikaterini Patsatsi, Dedee F. Murrell

**Affiliations:** ^1^Autoimmune Bullous Diseases Unit, 2nd Dermatology Department, Aristotle University School of Medicine, Thessaloniki, Greece; ^2^Department of Dermatology, St George Hospital, University of New South Wales, Sydney, NSW, Australia

**Keywords:** Bruton's tyrosine kinase, rilzabrutinib, BTK inhibition, Pemphigus vulgaris, pemphigus foliaceus

## Abstract

Bruton Tyrosine Kinase (BTK) has a key role in multiple pathways involved in inflammation and autoimmunity. Therefore, BTK has become a new therapeutic target for a group of hematologic and autoimmune disorders. The pharmaceutical industry has invested in the clinical development of BTK inhibitors during the last decade. Ibrutinib, for example, which was the first BTK inhibitor to be used in clinical trials, has two approved indications, mantle cell lymphoma and chronic lymphocytic leukemia, and remains under evaluation for additional indications. Rillzabrutinib (PRN1008) is a new, highly potent and selective inhibitor of BTK. Early studies performed in canine pemphigus demonstrated effectiveness. A proof-of-concept, multicenter, phase 2 trial has recently showed the efficacy and safety of oral rilzabrutinib in pemphigus vulgaris. In this mini review, we present evidence regarding the mechanisms affected by BTK inhibition and the concept of BTK inhibition as an emerging new treatment in pemphigus.

Bruton Tyrosine Kinase (BTK) has a key role in multiple pathways involved in inflammation and autoimmunity. Therefore, BTK has become a new therapeutic target for a group of hematologic and autoimmune disorders. The pharmaceutical industry has invested in the clinical development of BTK inhibitors during the last decade. Ibrutinib, for example, which was the first BTK inhibitor to be used in clinical trials, has two approved indications, mantle cell lymphoma and chronic lymphocytic leukemia, and remains under evaluation for additional indications (1).

In this mini review, we present evidence regarding the mechanisms affected by BTK inhibition and the concept of BTK inhibition as an emerging new treatment in pemphigus.

## What is BTK

Bruton tyrosine kinase (BTK) is an enzyme with a major role in both the innate and the adaptive immune responses. It is part of the signaling pathway of most white blood cells, apart from T cells and plasma cells. BTK contributes significantly in the proliferation and differentiation of B-cells. It also has a role in myeloid cell inflammatory cytokine production. Thus, BTK is being investigated nowadays as a promising target for the treatment of immunological disorders and B-cell hematological malignancies.

## Dr. Bruton

In 1952, the American pediatrician Dr. Ogden Bruton first described a case of an 8 year-old boy who presented with recurrent bacterial sepsis, osteomyelitis and otitis (2). The patient's failure to produce antibodies was attributed to the lack of a gamma globulin fraction. This was the first case of X- linked agammaglobulinemia (XLA), a disease manifested by markedly decreased serum immunoglobulins and almost total absence of B cells in the peripheral blood. The defect in XLA was initially mapped in 1986 (3). In 1993, it was found that XLA was due to mutations in the gene encoding a cytoplasmic tyrosine kinase which was named Bruton tyrosine kinase (4). It is estimated that 1 in 150,000 males is born with this BTK gene deficiency.

A less severe X-linked immunodeficiency has been described in mice. An important observation was that these mice were resistant to acquiring lupus (5) and collagen induced arthritis (6).

## Agammaglobulinaemia

X-linked agammaglobulinemia (XLA) is an immunodeficiency typically presented with a failure to produce mature B lymphocytes and accompanied with a failure of Ig heavy chain rearrangement. The defect in XLA resides in Bruton tyrosine kinase (BTK), also known as B cell progenitor kinase (BPK) or agammaglobulinemia tyrosine kinase (ATK), a key regulator in B-cell development (7). There is a genetic heterogeneity in XLA. Most cases are caused by mutation in the SH3 Domain Containing Kinase Binding Protein 1 (SH3KBP1) gene on chromosome Xq22.

## Family of Tyrosine Kinases

Tyrosine kinases are a large multigene protein family related to cancer and many diseases. More than 90 kinase genes have been identified in the human genome.

The tyrosine kinases are divided into two main classes, receptor and non-receptor tyrosine kinases.

The transmembrane receptor-linked kinases are 58 and distributed into 20 subfamilies. The non-receptor tyrosine kinases are 32 and placed in 10 subfamilies. This family of kinases function in multicellular pathways. Signals concerning cell differentiation, growth, adhesion, and death are transmitted through them and there is evidence that they play a role in cancer and in many diseases (8).

## B—Cell Malignancies and BTK Inhibition

BTK acts as an essential kinase of B-cell–receptor signaling, intercedes actions within the tumor microenvironment and enhances the proliferation and survival of malignant cells. BTK is a driving factor for the activation of various active pathways of malignant cell viability, including the AKT, extracellular signal–regulated kinase (ERK), and nuclear factor kappa light-chain enhancer of activated B cells (NF-κB) pathways (1, 7).

Ibrutinib, an oral covalent BTK inhibitor, has substantiated clinical efficacy in newly diagnosed as well as relapsed lymphoma patients compared to traditional chemotherapy (9). The first paradigm of therapeutic benefit targeting BTK was the rapid approval of ibrutinib by the US Food and Drug Administration (FDA) as a therapeutic option for chronic lymphocytic lymphoma/small lymphocytic lymphoma (CLL/SLL) and MCL (mantle cell lymphoma) in 2014 and 2013, respectively. It was subsequently approved as monotherapy in patients with lymphoplasmacytic lymphoma (LPL)/ WM (Waldenstrom's macroglobulinemia) and marginal zone lymphoma (MZL) in 2015 and 2017, respectively (10). Its safety profile has been a concern due to extended off-target kinase inhibition including Tec kinases, epidermal growth factor receptor (EGFR) and interleukin 2-inducible T cell kinase (ITK) (11). Associated side effects, such as atrial fibrillation, bleeding, infections, and arthralgias have led to treatment discontinuation in many patients with CLL.

The next generation of BTK inhibitors aimed to show less off-target inhibition and improved tolerability with comparable efficacy. Acalabrutinib, a selective covalent BTK inhibitor, was approved as a treatment option for CLL by the FDA in 2019 (12). Other next generation BTK inhibitors, such as zanubrutinib, tirabrutinib, vecabrutinib, and fenebrutinib, are currently studied (13).

## Pemphigus and Signaling Pathways

In pemphigus, in genetically susceptible individuals, an autoimmune reaction is driven by CD4 autoreactive T lymphocytes which are specific for Dsg molecules and induce the production of anti Dsg- antibodies by B lymphocytes (14).

In pemphigus vulgaris (PV) and pemphigus foliaceus (PF), target antigen specific autoantibodies induce keratinocyte detachment (acantholysis) and blister development.

Dsg1 is mainly expressed in the upper layers of the epidermis while Dsg3 is mostly expressed in the lower. The Dsg1/Dsg3 compensation theory explains why skin and mucosa are not equally affected by the target antigen specific autoantibodies in different pemphigus forms (15). In PV, for example, sera with Dsg3- specific IgG do not induce suprabasal acantholysis because Dsg1 is sufficiently expressed and compensates epidermal adhesion in the absence of Dsg3. Anti-Dsg3 IgG causes loss of adhesion in the mucous membranes as there is a low expression of Dsg1, which is insufficient to compensate disabled Dsg3 adhesion (15).

The exact cascade resulting in pemphigus acantholysis is still under research. Three major events following the binding of anti-Dsg IgG have been described. The direct interference with Dsg transinteraction through homophilic and heterophilic binding of Dsg molecules to Dsg and Dsc molecules on neighboring cells is a phenomenon termed steric hindrance. Dsg transinteraction via steric hindrance and altered outside-in-signaling by pemphigus autoantibodies leads to loss of desmosomal integrity and blister formation (14, 15).

Pemphigus is also known as a desmosome-remodeling disease because remodeling of Dsg expression on the cell surface results in internalization and depletion of Dsg. This depletion process of Dsg leads to acantholysis mainly induced by a direct interference of trans-interaction of Dsg. Desmosomes become fragile as they lose their adhesive properties at protein levels and are more susceptible for subsequent depletion processes (16).

Activation of keratinocyte intracellular signaling pathways is a crucial component of pemphigus IgG-mediated acantholysis. The signaling events impair the cytoskeletal architecture. These mechanisms don't apply equally for Dsg1 IgG- and Dsg3 IgG-binding. Upon targeting of Dsg1, Ca2+ influx is induced and therefore the extracellular-signal-regulated kinase (ERK) pathway is activated. On the other hand, after binding of Dsg3-specific IgG, signaling via p38MAPK occurs within the epidermis but not in mucosal tissues, and SRC family (non-receptor tyrosine kinases) and EGFR pathways are activated (17). According to the current literature, it is debated whether non-Dsg antibodies contribute to pemphigus phenotype (18–20).

In studies where PV Dsg-3 autoantibodies were used to initiate desmosome signaling in human keratinocyte cell cultures, it has been observed that PV IgG binding to Dsg3 activates desmosomal signal transduction cascades resulting in p38 mitogen-activated protein kinase (MAPK) and Heat Shock Protein—HSP27 phosphorylation and cytoskeletal reorganization. Both p38MAPK and HSP27 regulate components of the cytoskeleton including actin and intermediate filaments. These observations supported a mechanistic role for signaling in PV IgG-induced acantholysis. Consequently, it was suggested that targeting desmosome signaling via inhibition of p38MAPK and HSP27 phosphorylation may act as a novel targeted treatment for PV and other desmosome-related blistering diseases (21).

It was found also that PV IgG causes internalization of cell-surface Dsg3 into endosomes as early as 4 h. This cell-surface Dsg3 internalization and depletion was blocked by p38MAPK inhibition in pemphigus mouse models (22). The same authors demonstrated two peaks of p38MAPK activation in pemphigus keratinocyte tissue culture and mouse models. Examination of the temporal relationship of p38MAPK phosphorylation and apoptosis showed that apoptosis occurs at or after the second peak of p38MAPK activation. The ability of inhibitors of p38MAPK to block activation of the proapoptotic proteinase caspase-3 and the time course of p38MAPK activation and apoptotic markers suggest that activation of apoptosis is downstream to p38MAPK activation in pemphigus acantholysis. Therefore, blocking caspase-dependent Dsg degradation may augment cell-cell adherence and reduce the acantholytic effects of pathogenic IgG (23).

The activation of intracellular signaling events mediated by p38MAPK and HSP27 was also observed in pemphigus patient skin and was analogous to the increased phosphorylation of those two proteins (24). All the above autoantibody-triggered cellular signaling pathways are considered as pathogenic co-mechanisms in pemphigus but, none can solely induce acantholysis.

In pemphigus lesions dermal infiltrates contain interstitial and perivascular neutrophils and eosinophils evolving from innate immune response activation. Hence, not only adaptive but also innate immunological pathways may provide therapeutic targets.

The concept behind the employment of BTK inhibition in pemphigus is that a selective BTK inhibitor has the potential to focus on multiple pathways involved in autoimmunity, like modulation of BCR-mediated B-cell pathways, inhibition of FcεR-induced cytokine release from monocytes and macrophages, mediator release, neutrophil migration and FcγR-induced mast cell degranulation.

## Ibrutinib Report for a Paraneoplastic (PNP) Case

The effectiveness of BTK inhibition in pemphigus was first reported in a 51-year patient who suffered from chronic lymphocytic leukemia and developed paraneoplastic pemphigus (PNP). CLL disease control was achieved with ibrutinib and together there was a significant improvement of his pemphigus lesions. This was the first indication that ibrutinib may be added as a treatment option for pemphigus (25).

## Rilzabrutinib Biology

Rillzabrutinib (PRN1008) is a highly potent inhibitor of BTK with unique reversible covalent binding that has the potential to improve the safety profile compared with irreversible BTK inhibitors such as ibrutinib.

Rilzabrutinib binds in a covalent manner, enhancing selectivity by forming a chemical bond to a particular cysteine residue present in BTK. The addition of the covalent binding site increases the selectivity of rilzabrutinib for BTK vs. other kinases and reduces the dissociation rate to provide BTK binding dynamics that allow for convenient dosing. Reversible BTK binding allows rapid restoration of BTK function following rilzabrutinib withdrawal and reduces off-target effects (26).

A significant advantage of rilzabrutinib is that only short exposure is required to get a clinical benefit (27). Low systemic exposure reduces side effects, increases tolerability and promotes effectiveness and drug survival.

Rilzabrutinib acts on a range of immune cells. It inhibits B cell activation through inhibition of the BCR. However, it doesn't cause B cell depletion or cellular cytotoxicity.

This is a key difference from current therapies like rituximab because it doesn't provoke prolonged immune suppression. Additionally, rilzabrutinib rapidly inhibits Ab-mediated immune cell activation through Fc-receptor signaling. The activation of macrophages and monocytes via cross-linking of IgG and FcgR is a BTK dependent function; the ability of rilzabrutinib to prevent IgG mediated FcgR activation was illustrated in rodent models of lupus nephritis and immune thrombocytopenia (ITP). Rilzabrutinib has shown a unique dual Ab mechanistic approach over the standard treatments for immune-mediated diseases, based on its fast action on halting self-reactive Ab signaling and its longer-term impact on the formation of new autoantibodies (27).

Inhibition of BTK by rilzabrutinib also disrupts the signaling pathway for neutrophil recruitment. This alleviation of neutrophil recruitment may also be beneficial for patients with immune-mediated disorders, because of the reduced neutrophil—produced reactive oxygen species and associated tissue damage (28).

Rilzabrutinib does not affect the normal platelet aggregation and therefore, there is not an increased risk of bleeding as with irreversible BTKis, e.g., ibrutinib. Moreover, it isn't active on B cells which are stimulated via a non-BTK–mediated pathway or on T cells, keeping other receptors and signaling pathways that are vital for maintaining immunity, antiviral responses and lymphocyte counts in immune-compromised patients (27).

It is important that rilzabrutinib specifically suppresses inflammatory cellular activities in cells activated in immune-mediated diseases like mast cells, basophils and neutrophils without killing them. This action is fast, as evidenced by rilzabrutinib's rapid efficacy in a mouse model of immune thrombocytopenia (ITP) and in rats with collagen-induced arthritis (CIA) (27).

**Figure 1:**
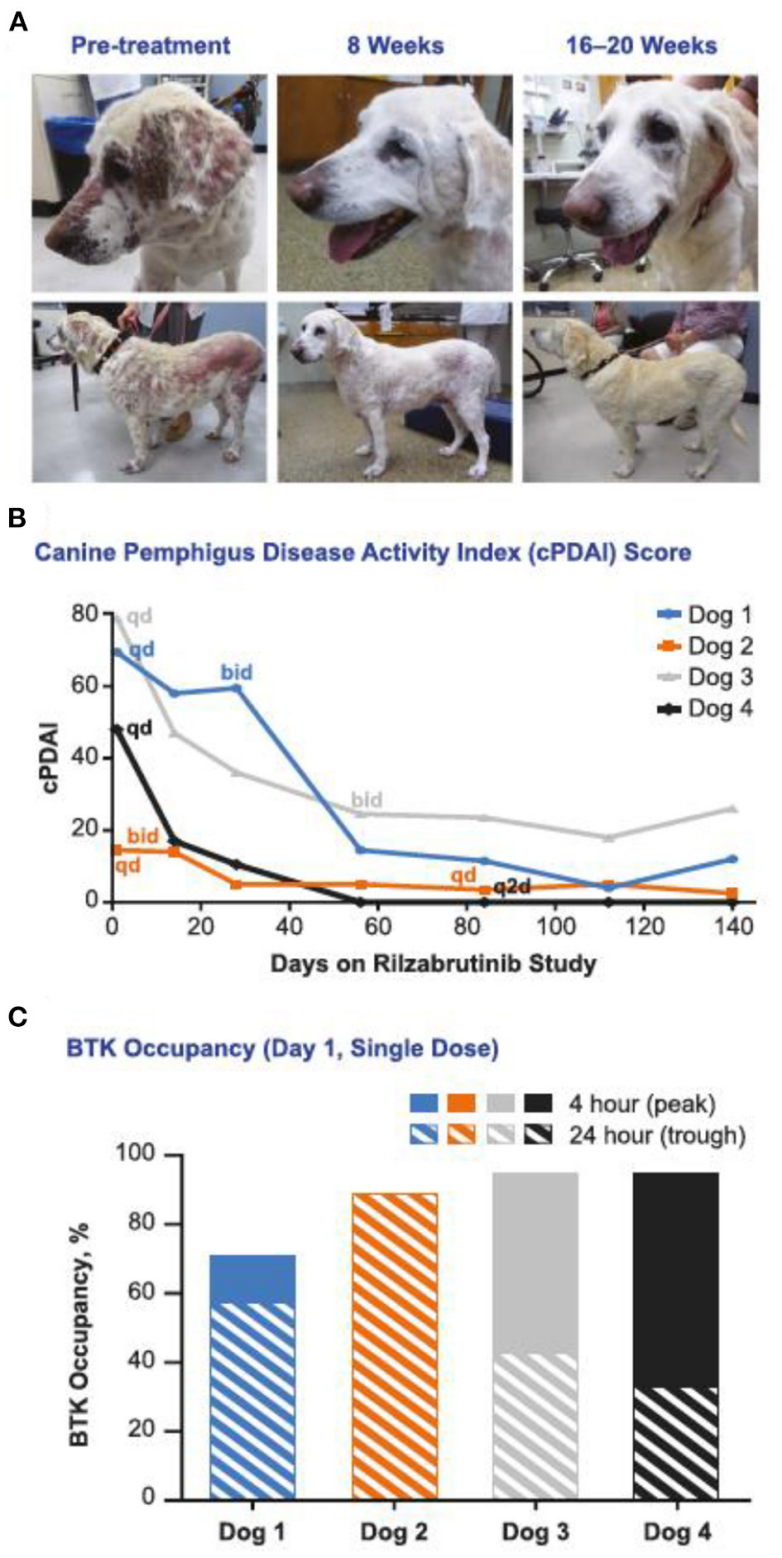
Rilzabrutinib monotherapy (without CSs) was effective for treatment of canines with naturally occurring PF. **(A)** a 13-y-old, yellow Labrador retriever with an initial cPDAI score of 69.5 began with a 500 mg/d dosage of rilzabrutinib and was escalated to 500 mg every 12 h at week 4. Significant decreases in lesions and regrowth of nearly all her coat was achieved by week 20. **(B)** cPDAI scores over time showed 77–100% improvement with rilzabrutinib dosing as monotherapy (*n* = 4 dogs). **(C)** BTK occupancy measurements ranged from 50 to 93% at the 4-h (peak) and 24-h (trough) timepoints [adopted by Langrish et al. (27)].

In a phase 1 study in 62 healthy volunteers, rilzabrutinib was well-tolerated following oral administration. No severe adverse events occurred during the trial. The most common adverse events were mild and mainly were associated with the gastrointestinal system (29). Another BTK inhibitor-PRN473 showed a promising response in canine pemphigus foliaceus (PF) but had lower bioavailability in human than PRN1008 (30). In a Phase II canine pemphigus foliaceus study with PRN1008, all dogs showed reduction in lesions and canine PDAI score during the first 2 weeks of treatment and continued to achieve near complete remission by 20 weeks (31). Rilazabrutinib was granted Orphan Drug Designation by FDA for the treatment of patients with PV, after the encouraging results of a phase II open-label cohort study examining rilzabrutinib in adult patients with PV (Figure 1) (32).

The results of this proof-of-concept, multicenter, phase 2 trial examining the efficacy and safety of oral rilzabrutinib in PV were published recently (33). Rilzabrutinib alone, or in combination with low CS doses was safe with fast clinical response in PV patients (33). Following this proof-of-concept trial, a phase 3 pivotal study (PEGASUS; NCT03762265) of rilzabrutinib vs. placebo with CS taper is underway for PV.

## Conclusion

BTK inhibitors offer a new paradigm for the treatment of autoimmune diseases and AIBD in particular, with pemphigus having the most compelling unmet need, since they potentially act much faster via the innate immune system, compared with rituximab. Early studies performed in canine pemphigus demonstrated effectiveness. A proof-of-concept, multicenter, phase 2 trial recently showed the efficacy and safety of oral rilzabrutinib in pemphigus vulgaris and a large phase III RCT of rilzabrutinib vs. placebo is currently underway.

## Author Contributions

All authors contributed in conception, literature search, and writing.

## Conflict of Interest

AP reports honoraria as a speaker/advisor/investigator from abbvie, Jannsen, Leo Pharma, Pharmaserv—Lilly, Genesis Pharma, Novartis, Pfizer, Principia, Sanofi, UCB. DM reports advisory board and principal investigator/investigator roles for Principia Biopharma and Roche; principal investigator for clinical studies/advisor supported by AbbVie, Amgen, ArgenX, AstraZeneca, Botanix, Dermira, Eli Lilly, Galderma, Janssen, Leo, Lilly, Novartis, Pfizer, Regeneron, Sanofi, Sun Pharma, and UCB; patent for EB with topical sirolimus.

## Publisher's Note

All claims expressed in this article are solely those of the authors and do not necessarily represent those of their affiliated organizations, or those of the publisher, the editors and the reviewers. Any product that may be evaluated in this article, or claim that may be made by its manufacturer, is not guaranteed or endorsed by the publisher.
